# The diagnostic value of ultrasonography-derived edema of the temporal artery wall in giant cell arteritis: a second meta-analysis

**DOI:** 10.1186/1471-2474-11-44

**Published:** 2010-03-08

**Authors:** Aikaterini Arida, Miltiades Kyprianou, Meletios Kanakis, Petros P Sfikakis

**Affiliations:** 1 First Dept. of Propedeutic and Internal Medicine, Laikon Hospital, Athens University Medical School, Athens, Greece

## Abstract

**Background:**

Ultrasonography of temporal arteries is not commonly used in the approach of patients with suspected giant cell arteritis (GCA) in clinical practice. A meta-analysis of primary studies available through April 2004 concluded that ultrasonography could indeed be helpful in diagnosing GCA. We specifically re-examined the diagnostic value of the ultrasonography-derived halo sign, a dark hypoechoic circumferential thickening around the artery lumen, indicating vasculitic wall edema, in GCA.

**Methods:**

Original, prospective studies in patients with suspected GCA that examined ultrasonography findings of temporal arteries using the ACR 1990 classification criteria for GCA as reference standard, published through 2009, were identified. Only eight studies involving 575 patients, 204 of whom received the final diagnosis of GCA, fulfilled technical quality criteria for ultrasound. Weighted sensitivity and specificity estimates of the halo sign were assessed, their possible heterogeneity was investigated and pooled diagnostic odds ratio was determined.

**Results:**

Unilateral halo sign achieved an overall sensitivity of 68% (95% CI, 0.61-0.74) and specificity of 91% (95% CI, 0.88-0.94) for GCA. The values of inconsistency coefficient (I^2^) of both sensitivity and specificity of the halo sign, showed significant heterogeneity concerning the results between studies. Pooled diagnostic odds ratio, expressing how much greater the odds of having GCA are for patients with halo sign than for those without, was 34 (95% CI, 8.21-138.23). Diagnostic odds ratio was further increased to 65 (95% CI, 17.86-236.82) when bilateral halo signs were present (sensitivity/specificity of 43% and 100%, respectively). In both cases, it was found that DOR was constant across studies.

**Conclusion:**

Temporal artery edema demonstrated as halo sign should be always looked for in ultrasonography when GCA is suspected. Providing that currently accepted technical quality criteria are fulfilled, halo sign's sensitivity and specificity are comparable to those of autoantibodies used as diagnostic tests in rheumatology. Validation of revised GCA classification criteria which will include the halo sign may be warranted.

## Background

According to the American College of Rheumatology (ACR) 1990 criteria for the classification of vasculitis, diagnosis of temporal or giant-cell arteritis (GCA), the most common form of systemic vasculitis in adults, is based on clinical grounds and the result of a temporal artery biopsy. The presence of three or more of the following five criteria, (1) age of 50 years and older, (2) new onset of localized headache, (3) temporal artery tenderness on palpation or decreased pulsation, (4) erythrocyte sedimentation rate of 50 mm/h and higher, (5) abnormal temporal artery biopsy, yields a sensitivity of 93.5% and a specificity of 91.2% for the diagnosis of GCA when compared to other vasculitides [1,2]. Because of the possible development of severe ischemia, i.e. visual impairment with loss of vision occurring in almost 15% of patients, early diagnosis of GCA and prompt initiation of treatment is mandatory. Certainty about the correct diagnosis is imperative in view of the potentially important adverse affects of the required long-term corticosteroid treatment. Since the most specific findings in patients' history, physical examination and routine laboratory testing have sensitivities of about 50%, a temporal artery biopsy has traditionally been recommended [2].

In 1997, Schmidt and colleagues were the first to examine the use of ultrasonography of the temporal arteries in the diagnosis of GCA [3]. When applied to inflamed temporal arteries, ultrasonography may show, a) edema, referred to as halo sign, indicated by a dark hypoechoic circumferential wall thickening around the artery lumen which disappears after corticosteroid treatment within 2-3 weeks, b) stenoses, expressed by segmental increases of blood flow velocity, and, c) occlusions, expressed by the absence of flow in the temporal artery (on color or power doppler ultrasonography). During the last decade ultrasonography has attracted considerable interest as a non-invasive diagnostic tool for patients suspected of having GCA. A meta-analysis of primary studies available through April 2004 [4] concluded that halo sign, stenoses and occlusions demonstrated by ultrasonography could be indeed helpful in diagnosing GCA. On the other hand, in an elegant study published in 2002, Salvarani et al stated that ultrasonography is not better than a careful physical examination for the detection of a biopsy positive GCA [5].

In a more recent controlled study performed in our center [6], it was found that blood flow alterations (stenoses and/or occlusions) demonstrated in temporal arteries with doppler ultrasonography are neither specific nor sensitive for GCA, since these findings were equally common among elderly individuals or patients with macrovascular disease associated with diabetes mellitus or stroke, due to the temporal artery atherosclerotic changes. The presence of unilateral halo alone yielded 82% sensitivity and 91% specificity for GCA in our study, while the specificity reached 100% when halos were found bilaterally. At follow-up ultrasonography examinations performed at 2 and 4 weeks following initiation of corticosteroid treatment for GCA, halos disappeared in all patients [6]. To further explore the utility of ultrasonography in patients with clinically suspected GCA, we examined the specific value of the halo sign for GCA diagnosis in a meta-analysis which includes only prospective studies that meet technical quality criteria for ultrasound [7,8].

## Methods

We conducted an extensive Medline/PubMed, EMBASE and Cochrane databases search for original primary studies published in any language through December 2009 that examined the sensitivity and specificity of the halo sign demonstrated by temporal artery ultrasonograply for GCA diagnosis versus the ACR 1990 criteria for the classification of vasculitis, used as a reference standard [1]. The search strategy was based on combination of index terms: giant cell arteritis, temporal arteritis, vasculitis, ultrasonography, duplex, Doppler, ultrasound, diagnosis. Primary studies which did not examine the diagnostic value of the halo sign independently of concomitant blood flow abnormalities were not included in this meta-analysis. We analysed only prospective studies which enrolled patients with clinically suspected GCA, including patients who had initially received the diagnosis of polymyalgia rheumatica. All studies used color doppler or duplex ultrasonography with appropriate color intensity and doppler settings [7]. The temporal arteries were examined in both longitudinal and transverse plane. Because there is substantial variability in equipment from machine to machine, from manufacturer to manufacturer, and between older and newer ultrasound equipment, only studies that used industry-wide standards for Doppler measurement were included [7,8], in contrast to the previous meta-analysis in which studies were eligible regardless of the ultrasound quality method used [4].

### Statistical analysis

Sensitivity and specificity estimates of the halo sign together with their 95% confidence intervals were calculated for each study and for the total sample size of studies. Two meta-analytic methods of weighted independent estimation of overall sensitivity and specificity were applied to overcome biased estimation of such calculations; namely, the fixed effect model of Mantel-Haenzel weights each study by the inverse of its variance [9], whereas the random effects model by DerSimonian-Laird also incorporates between study variation and it usually yields wider confidence intervals [10], thus is preferable in a proven heterogeneity between studies. The heterogeneity of sensitivity and specificity between studies was evaluated using Cochran's Q and chi-square-test, followed by the calculation of inconsistency (I^2^). The I^2 ^statistic is calculated from Q and can be interpreted as the percentage variability in study results attributable to between-study differences rather than chance [11]. The degree of variability among results of different studies was also evaluated graphically by plotting the sensitivity and specificity from each study on a forest plot.

Because the sensitivity and specificity are by their nature dependent on each other, a more appropriate analysis, which takes into account their independence, such as the summary receiver-operating characteristic (sROC) curve analysis was also applied. The sROC curve is well established as a method of summarizing the performance of a diagnostic test [12]. The area under the ROC curve (AUC) and the index Q* are useful summary measures: AUC is maximized when the study odds ratio are homogeneous (equals with 1 for a perfect test), whereas Q* is invariant to heterogeneity [13,14]. Moreover, the diagnostic odds ratio (DOR), an additional measure of the test accuracy, useful in meta-analysis, was determined [15]. The DOR expresses how much greater the odds of having the disease are for a subject with a positive test result than for a subject with a negative test result.

Since an important extra source of variation in meta-analysis of diagnostic accuracy relates to the fact that different studies may have used, explicitly or implicitly, different thresholds to define positive and negative test results, Spearman correlation coefficient between the sensitivity and specificity was used (if the threshold effect exists an inverse correlation appears). A visual cue to the possible existence of this source of variation can be given by the plot the sensitivity and specificity on a ROC plane. If such a threshold effect exists, the points will show a curvilinear pattern. This hypothesis can be tested using Moses' regression model, both with unweighted and weighted, with inverse variance. If the slope (b) of the regression equation is not significantly different from zero, then we can assume that DOR is constant and that there is no heterogeneity between the studies.

## Results

Of a total of 16 primary studies that examined the diagnostic value of the halo sign demonstrated by temporal artery ultrasonograply versus the ACR 1990 criteria for the classification of vasculitis, used as a reference standard [3,5,6,16-28], only 9 studies met our inclusion/exclusion criteria [3,5,6,17-19,21,24,27]. To avoid duplicate reports on the same patients, one study [3] was excluded. Collectively, the 8 remaining studies, from 7 centers, involved 575 different patients (each of them reported once). These patients had suspected GCA and underwent temporal artery ultrasonography before biopsy. Of them, 204 received a final diagnosis of GCA diagnosis on the basis of 3 or more of the ACR classification criteria [1]

**Figure 1:**
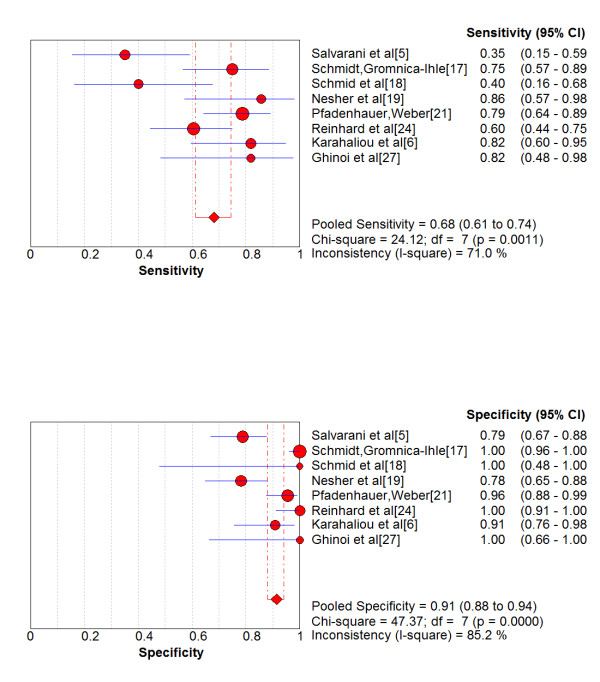
**Forest plot of the sensitivity and specificity of the temporal artery ultrasonography- derived halo sign compared to final diagnosis of giant-cell arteritis in patients with suspected disease**.

As shown in Figure 1, the presence of unilateral halo sign achieved an overall sensitivity of 68% and an overall specificity of 91% compared with final diagnosis. The area under the sROC curve (AUC) was 0.87 (SE = 0.09) with a Q* point value, denoting the diagnostic at which the correct diagnosis is constant for all subjects, of 0.80 (95% CI 0.62-0.97) (SE = 0.0883).

**Figure 2:**
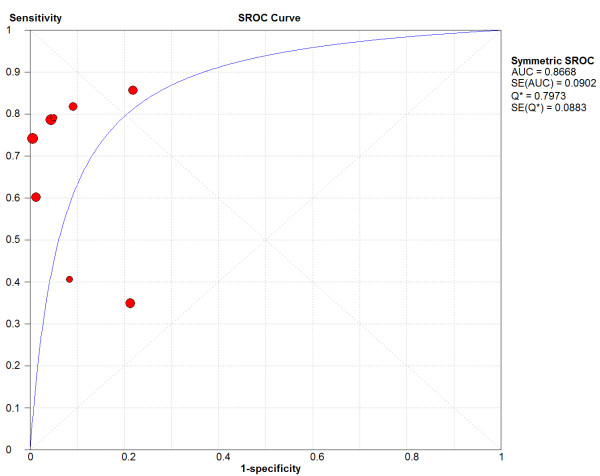
**Summary receiver-operating characteristic (sROC) curves of the temporal artery ultrasonography- derived halo sign compared to final diagnosis of giant-cell arteritis in patients with suspected disease**.

Notably, there was significant heterogeneity between the studies in terms of sensitivity (chi-square test and inconsistency were 24.12, p < 0.001 and 71%, respectively), as well as in terms of specificity (chi-square test and inconsistency were 47.37, p < 0.001 and 85.2%, respectively). As also shown by the sROC curves depicted in Figure 2, there was a significant variation concerning the diagnostic thresholds between studies to define positive and negative results. The Spearman correlation coefficient of sensitivity with specificity was 0.238 (p = 0.570). Likewise, both unweighted and weighted Moses' models showed that the slope (b) of the regression equation did not differ from zero, implying a constant DOR and no heterogeneity between studies.

**Figure 3:**
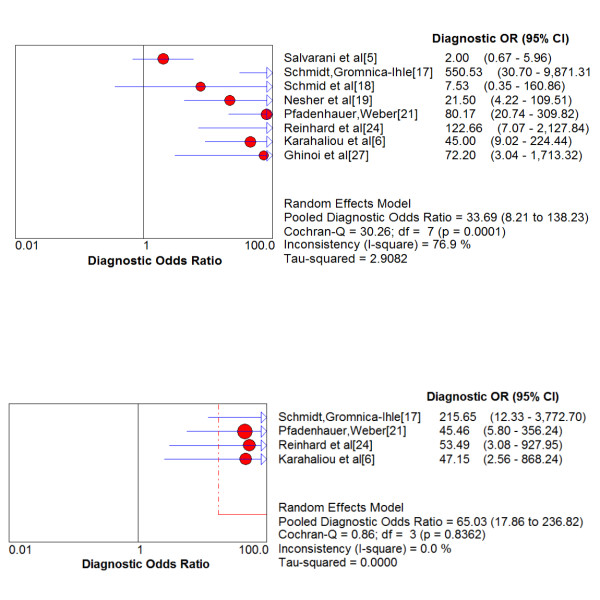
**Diagnostic Odds Ratios of the temporal artery ultrasonography- derived unilateral (upper panel) and bilateral (lower panel) halo sign compared to final diagnosis of giant-cell arteritis in patients with suspected disease**.

As shown in Figure 3, the pooled DOR of the unilateral halo sign for GCA was equal to 33.69 (95% CI = 8.21-138.23). The discrimination power (ln(OR)) was 3.52 (95% CI 2.11-4.93).

Finally, 4 studies, including a total of 380 patients provided data concerning the presence of bilateral halo in CDS [6,17,21,24]. Indeed, 62 of 63 patients with bilateral halo were diagnosed with GCA resulting in a specificity of 100%, whereas sensitivity was 43%. As shown in Figure 3, pooled DOR of bilateral halo reached 65.03 (95% CI = 17.86-236.82). The discrimination power (ln(OR)) was 4.17 (96% CI 2.88-5.47).

## Discussion and Conclusions

Our meta-analysis revealed a sensitivity of 68% and a specificity of 91% for the unilateral halo sign, as well as 43% and 100%, respectively, for the bilateral halo sign in temporal artery ultrasonography for GCA diagnosis, when the 1990 ACR criteria are used as the reference standard. Possible limitations of these criteria have been discussed in the literature [29], but revision(s) are not available to date. In the first meta-analysis by Karassa et al, assessing the test-performance of ultrasonography for GCA in studies published up to April 2004, the sensitivity and specificity of the halo sign versus ACR criteria (without referring to unilateral or bilateral presence) were reported to be 55% and 94%, respectively [4]. The considerably higher sensitivity of the unilateral halo reported herein, in addition to the inclusion of 2 recent studies [6,27], is most probably due to significant quality differences among studies collectively analysed in the previous meta-analysis without applying established technical quality criteria for ultrasound. We avoided presenting an additional analysis of how the presence of blood flow abnormalities (stenoses/occlusions) would further increase the sensitivity of ultrasonography because very limited information on the coexistence of halo sign with such abnormalities, which are common findings in elderly patients without GCA [6], was provided in relevant studies.

We restricted our analysis to studies using the ACR criteria as reference standard. Those studies focusing only on comparison between ultrasonography and temporal artery biopsy findings [30-38] were not analysed. In our opinion, such a comparison could be misleading given the fact that biopsy has a high probability of false negative results because of the segmental nature of the disease, and thus cannot exclude the diagnosis of vasculitis when negative [39-41]. For example, in a recent study, 19% of patients with suspected GCA and a negative temporal artery biopsy were eventually diagnosed as GCA. Diagnosis was established on the basis of at least 3 of the remaining ACR classification criteria, improvement of symptoms within 3 days of corticosteroid therapy and the absence of other condition relevant to the patients' symptoms during a 6-month follow-up [42]. A similar percentage of 19% of patients with GCA had negative biopsy results in a cohort of 271 patients from another center [43]. Moreover, as a recent study suggests, up to 13% of patients with GCA could have been misdiagnosed as biopsy-negative had a biopsy been done only unilaterally [44], which is the case in the vast majority of patients included in all relevant studies. Finally, in 8 of 9 studies analysed herein, the presence of halo sign in ultrasonography was used to direct temporal artery biopsy, clearly leading to an underestimation of the true diagnostic performance of the halo sign.

A sensitivity of 68% and a specificity of 91% for the unilateral halo sign in temporal artery ultrasonography for GCA diagnosis, when the ACR criteria are used as the reference standard, are comparable to sensitivity and specificity estimates of other diagnostic tests that are widely used in rheumatology. For example, in a meta-analysis on the diagnostic value of autoantibody measurements in rheumatoid arthritis, the sensitivity and specificity of rheumatoid factor were 69% and 85%, respectively, whereas of anti-cyclic citrullinated peptide were 67% and 95% [45]. Moreover, anti-dsDNA antibodies measured by commercially available ELISA have sensitivities and specificities of 61% and 95%, respectively, for systemic lupus erythematosus [46]. Both rheumatoid factor and anti-dsDNA antibodies are included in the sets of ACR classification criteria for the respective diseases.

Interestingly, the values of inconsistency coefficient (I^2^) of both sensitivity and specificity of the halo sign, showed significant heterogeneity concerning the results between studies, probably caused by a threshold effect. It is difficult to assume that this threshold effect is attributable only to ultrasonography findings *per se*. Other contributing factors may be differences in clinical diagnostic skills or biopsy method, and/or interpretation of histology results across studies. In addition, referral of patients for ultrasonography by physicians non-specialised to connective tissue diseases, i.e. internists, general practitioners, ophthalmologists, or neurologists may lead to a low pretest probability for positive GCA in some studies. Such factors may clearly result to variability between studies concerning the diagnostic thresholds.

Because of the trade-off nature of sensitivity and specificity, meta-analysis of diagnostic tests using these conventional expressions offers statistical challenges. Simple pooling of sensitivity and specificity may be not the most appropriate approach, as it ignores threshold differences. In addition, heterogeneity may lead to an underestimation of a test's performance. More recent evidence suggests that a more important index to define the accuracy of a given test result as a diagnostic tool is the pooled DOR [15]. The DOR offers considerable advantages in meta-analysis of diagnostic studies as it combines results from different studies into summary estimates with increased precision. In the present meta-analysis the pooled DOR of the unilateral halo sign was as high as 34. When bilateral halo signs were present, pooled DOR was higher than 65, indicating a considerable diagnostic accuracy of ultrasonography for GCA. Moreover, in both cases, it was found that DOR was constant across studies.

The results presented herein confirm that the halo sign in ultrasonography is useful in diagnosing GCA. A previously proposed algorithm [4] suggests that after a careful clinical examination and assessment of relevant laboratory data temporal artery ultrasonography examination should precede the biopsy in patients with suspected GCA, whereas among the various abnormalities which can be found in ultrasonography, only the halo sign should be considered. In case of bilateral halo signs, treatment could be initiated without proceeding with biopsy. If unilateral halos are present, a decision of directional biopsy is justified [4]. The results of the present meta-analysis further substantiate this algorithm. Moreover, in a recent study involving 182 patients from a single center, albeit not using the ACR criteria as the reference standard, it was found that GCA can be accurately diagnosed or excluded by ultrasonography, without biopsy, in two thirds of patients [28].

Comparing to temporal artery biopsy, ultrasonography is very well tolerated, more accessible, less costly and a more rapid and easier to perform procedure, with high reproducibility. An additional important advantage of ultrasonography is that full-length examination of the superficial temporal and other cranial arteries is allowed. Experts in the field suggest that certain parameters should be fulfilled for a correct US examination of the temporal arteries; namely, high-quality color Doppler US equipment with a linear probe that exhibits a frequency of more than 8 MHz, adequate experience with vascular US, knowledge of the US image of a normal temporal artery, and standardized US machine adjustments [47]. The color signal should cover the artery lumen exactly, not extend over parts of the lumen or cover only the center of the lumen. Along these lines, a sonographer should have investigated at least 50 persons without temporal arteritis to acquire relevant experience on the appearance of a normal temporal artery before assessing patients with suspected GCA [47].

The present meta-analysis, however, is by design unable to show a direct superiority of ultrasonography to microscopic examination of temporal artery biopsy, which might have some additional prognostic importance. As recently demonstrated in a study of 391 biopsy specimens from patients with GCA, the presence of giant cells was strongly associated with permanent visual loss, the most feared disease complication [48] On the other hand, studies have shown that ultrasound of the axillary arteries could increase the diagnostic yield for large vessel GCA, as characteristic findings, such as hypoechoic wall thickening and/or stenosis, are present in the vast majority of patients [49]. Finally, with recent and ongoing progress in probe technology, with gradually higher frequencies and matrix technology, sensitivities of the halo sign higher than 68% might be expected [50].

To conclude, temporal artery edema demonstrated as halo sign in ultrasonography, providing that technical quality criteria are fulfilled [51], should be always looked for in patients with suspected GCA. The diagnostic accuracy of the halo sign for GCA seems to be comparable to anti-cyclic citrullinated peptide antibody and rheumatoid factor for rheumatoid arthritis, or to anti-dsDNA antibody for systemic lupus erythematosus. The findings of this meta-analysis suggest that validation of a revised set of classification criteria for GCA which will include the halo sign is warranted.

## Abbreviations

GCA: giant-cell arteritis; ACR: American College of Rheumatology; DOR: diagnostic odds ratio; sROC: summary receiver operating characteristic; AUC: area under the ROC curve.

## Competing interests

The authors declare that they have no competing interests.

## Authors' contributions

A.A. participated in the design of the study, collected and analysed the data and helped to draft the manuscript. M. Kanakis participated in the design, collection and interpretation of data. M. Kyprianou performed the statistical analysis and participated in interpretation of data. P.P.S. conceived of the study and drafted the manuscript. All authors read and approved the final manuscript.

## Pre-publication history

The pre-publication history for this paper can be accessed here:

http://www.biomedcentral.com/1471-2474/11/44/prepub

## References

[B1] Hunder GG Arend WP Bloch DA Calabrese LH Fauci AS Fries JF Leavitt RY Lie JT Lightfoot RW Jr Masi AT The American College of Rheumatology 1990 criteria for the classification of vasculitis Arthritis Rheum 1990 33 1065 7 2390119 10.1002/art.1780330802

[B2] Klippel JH Stone JH Crofford LJ White PH Primer of Rheumatic Diseases 2008 13 New York: Springer Science+Business Media

[B3] Schmidt WA Kraft HE Vorpahl K Volker L Gromnica-Ihle EJ Color duplex ultrasonography in the diagnosis of the temporal arteritis N Engl J Med 1997 337 1336 42 10.1056/NEJM199711063371902 9358127

[B4] Karassa FB Matsagas MI Schmidt WA Ioannidis JP Meta-analysis: test performance of ultrasonography for giant-cell arteritis Ann Intern Med 2005 142 359 69 15738455 10.7326/0003-4819-142-5-200503010-00011

[B5] Salvarani C Silingardi M Ghirarduzzi A Lo Scocco G Macchioni P Bajocchi G Vinceti M Cantini F Ioni I Boiardi L Is duplex ultrasonography useful for the diagnosis of giant-cell arteritis? Ann Intern Med 2002 137 232 8 12186513 10.7326/0003-4819-137-4-200208200-00006

[B6] Karahaliou M Vaiopoulos G Papaspyrou S Kanakis MA Revenas K Sfikakis PP Colour duplex sonography of temporal arteries before decision for biopsy: a prospective study in 55 patients with suspected giant cell arteritis Arthritis Res Ther 2006 8 R116 10.1186/ar2003 16859533 PMC1779378

[B7] Schmidt WA Doppler Ultrasonography in the diagnosis of giant cell arteritis Clin Exp Rheumatol 2000 18 4 Suppl 20 S40 2 10948760

[B8] Grant EG Benson CB Moneta GL Alexandrov AV Baker JD Bluth EI Carroll BA Eliasziw M Gocke J Hertzberg BS Katanick S Needleman L Pellerito J Polak JF Rholl KS Wooster DL Zierler RE Carotid artery stenosis: gray-scale and Doppler US diagnosis--Society of Radiologists in Ultrasound Consensus Conference Radiology 2003 229 340 6 10.1148/radiol.2292030516 14500855

[B9] Mantel N Haenszel W Statistical aspects of the analysis of data from retrospective studies of disease J Natl Cancer Inst 1959 22 719 48 13655060

[B10] Fleiss JL The statistical basis of meta-analysis Stat Methods Med Res 1993 2 121 45 10.1177/096228029300200202 8261254

[B11] Higgins JPT Thomson SG Dees Deeks JJ Altman DG Measuring Inconsistency in meta-analysis BMJ 2003 327 557 60 10.1136/bmj.327.7414.557 12958120 PMC192859

[B12] Walter SD Properties of the summary receiver operating characteristic (SROC) curve for diagnostic test data Stat Med 2002 21 1237 56 10.1002/sim.1099 12111876

[B13] Moses LE Shapiro D Littenberg B Combining independent studies of a diagnostic test into a summary ROC curve: data-analytic approaches and some additional considerations Stat Med 1993 12 1293 316 8210827 10.1002/sim.4780121403

[B14] Littenberg B Moses LE Estimating diagnostic accuracy from multiple conflicting reports: a new meta-analytic method Med Decis Making 1993 13 313 21 10.1177/0272989X9301300408 8246704

[B15] Glas AS Lijmer JG Prins MH Bonsel GJ Bossuyt PM The diagnostic odds ratio: a single indicator of test performance J Clin Epidemiol 2003 56 1129 35 10.1016/S0895-4356(03)00177-X 14615004

[B16] Ihara M Yanagihara C Takeda N Hashimoto K Nishimura Y The significance of color duplex ultrasonography for the diagnosis of temporal arteritis Rinsho Shinkeigaku 1999 39 1001 5 10655758

[B17] Schmidt WA Gromnica-Ihle E Incidence of temporal arteritis in patients with polymyalgia rheumatica: a prospective study using colour Doppler ultrasonography of the temporal arteries Rheumatology (Oxford) 2002 41 46 52 10.1093/rheumatology/41.1.46 11792879

[B18] Schmid R Hermann M Yannar A Baumgartner RW Color duplex ultrasound of the temporal artery: replacement for biopsy in temporal arteritis Ophthalmologica 2002 216 16 21 10.1159/000048291 11901283

[B19] Nesher G Shemesh D Mates M Sonnenblick M Abramowitz HB The predictive value of the halo sign in color Doppler ultrasonography of the temporal arteries for diagnosing giant cell arteritis J Rheumatol 2002 29 1224 6 12064840

[B20] Schmidt WA Gromnica-Ihle E Duplex ultrasonography in temporal arteritis Ann Intern Med 2003 138 609 author reply 609-10 12667040 10.7326/0003-4819-138-7-200304010-00026

[B21] Pfadenhauer K Weber H Present state of ultrasonographic diagnosis of temporal arteritis. Results of a prospective study Nervenarzt 2003 74 683 90 10.1007/s00115-003-1532-3 12904870

[B22] Pfadenhauer K Weber H Giant cell arteritis of the occipital arteries--a prospective color coded duplex sonography study in 78 patients J Neurol 2003 250 844 9 10.1007/s00415-003-1104-2 12883928

[B23] Pfadenhauer K Weber H Duplex sonography of the temporal and occipital artery in the diagnosis of temporal arteritis. A prospective study J Rheumatol 2003 30 2177 81 14528514

[B24] Reinhard M Schmidt D Hetzel A Color-coded sonography in suspected temporal arteritis-experiences after 83 cases Rheumatol Int 2004 24 340 6 10.1007/s00296-003-0372-6 14600785

[B25] Vianna RN Mansour M Ozdal PC Souza Filho JP Bakalian S Saraiva VS Deschenes J Burnier MN Jr The role of ultrasound biomicroscopy in predicting the result of temporal artery biopsy in temporal arteritis patients: a preliminary study Eur J Ophthalmol 2005 15 655 9 28221431 10.5301/EJO.2008.1913

[B26] Houtman P Doorenbos B Dol J Bruyn G Doppler ultrasonography to diagnose temporal arteritis in the setting of a large community hospital Scand J Rheumatol 2008 37 316 8 10.1080/03009740801998804 18612936

[B27] Ghinoi A Zuccoli G Nicolini A Pipitone N Macchioni L Bajocchi GL Nicoli F Silingardi M Catanoso MG Boiardi L Salvarani C 1 T magnetic resonance imaging in the diagnosis of giant cell arteritis: comparison with ultrasonography and physical examination of temporal arteries Clin Exp Rheumatol 2008 26 3 Suppl 49 S76 80 18799059

[B28] Stammler F Grau C Schnabel A Value of colour Doppler ultrasonography in relation to clinical pretest probability in giant cell (temporal) arteritis Dtsch Med Wochenschr 2009 134 2109 15 10.1055/s-0029-1241899 19809960

[B29] Rao JK Allen NB Pincus T Limitations of the 1990 American College of Rheumatology classification criteria in the diagnosis of vasculitis Ann Intern Med 1998 129 345 52 9735061 10.7326/0003-4819-129-5-199809010-00001

[B30] Wenkel H Michelson G Correlation of ultrasound biomicroscopy with histological findings in diagnosis of giant cell arteritis Klin Monatsbl Augenheilkd 1997 210 48 52 10.1055/s-2008-1035013 9206734

[B31] Venz S Hosten N Nordwald K Lemke AJ Schröder R Böck JC Hartmann CF Felix R Use of high resolution color Doppler sonography in diagnosis of temporal arteritis Rofo 1998 169 605 8 9930213 10.1055/s-2007-1015349

[B32] Stammler F Ysermann M Mohr W Kuhn C Goethe S Value of color-coded duplex ultrasound in patients with polymyalgia rheumatica without signs of temporal arteritis Dtsch Med Wochenschr 2000 125 1250 6 10.1055/s-2000-7853 11098235

[B33] Roters S Szurman P Engels BF Brunner R The suitability of the ultrasound biomicroscope for establishing texture in giant cell arteritis J Ophthalmol 2001 85 946 8 10.1136/bjo.85.8.946 PMC1724073 11466252

[B34] LeSar CJ Meier GH DeMasi RJ Sood J Nelms CR Carter KA Gayle RG Parent FN Marcinczyk MJ The utility of color duplex ultrasonography in the diagnosis of temporal arteritis J Vasc Surg 2002 36 1154 60 10.1067/mva.2002.129648 12469046

[B35] Schmidt D Hetzel A Reinhard M Auw-Haedrich C Comparison between color duplex ultrasonography and histology of the temporal artery in cranial arteritis (giant cell arteritis) Eur J Med Res 2003 8 1 7 12578748

[B36] Murgatroyd H Nimmo M Evans A MacEwen C The use of ultrasound as an aid in the diagnosis of giant cell arteritis: a pilot study comparing histological features with ultrasound findings Eye 2003 17 415 9 10.1038/sj.eye.6700350 12724706

[B37] Romera-Villegas A Vila-Coll R Poca-Dias V Cairols-Castellote MA The role of color duplex sonography in the diagnosis of giant cell arteritis J Ultrasound Med 2004 23 1493 8 15498914 10.7863/jum.2004.23.11.1493

[B38] Zaragozá García JM Plaza Martínez A Briones Estébanez JL Martínez Parreño C Gómez Palonés FJ Ortiz Monzón E Value of the Doppler-ultrasonography for the diagnosis of temporal arteritis Med Clin (Barc) 2007 129 451 3 10.1157/13111003 17953909

[B39] Davies C Frost B Eshan O McLain AD Shandall A Temporal artery biopsy... who needs one? Postgrad Med J 2006 82 476 8 10.1136/pgmj.2005.043646 16822927 PMC2563776

[B40] Chong EW Robertson AJ Is temporal artery biopsy a worhtwile procedure? ANZ J Surg 2005 75 388 91 10.1111/j.1445-2197.2005.03399.x 15943722

[B41] Alberts MS Mosen DM Diagnosing temporal arteritis: duplex vs. biopsy QJM 2007 100 785 9 10.1093/qjmed/hcm103 18089544

[B42] Breuer GS Nesher R Nesher G Negative temporal artery biopsies: eventual diagnoses and features of patients with biopsy-negative giant cell arteritis compared to patients witout arteritis Clin Exp Rheumatol 2008 26 1103 6 19210879

[B43] Liozon E Loustaud V Fauchais AL Soria P Ly K Ouattara B Rhaiem K Nadalon S Vidal E Concurrent temporal (giant cell) arteritis and malignancy: report of 20 patients with review of the literature J Rheumatol 2006 33 1606 14 16832846

[B44] Breuer GS Nesher G Nesher R Rate of discordant findings in bilateral temporal artery biopsy to diagnose giant cell arteritis J Rheumatol 2009 36 794 6 10.3899/jrheum.080792 19228656

[B45] Nishimura K Sugiyama D Kogata Y Tsuji G Nakazawa T Kawano S Saigo K Morinobu A Koshiba M Kuntz KM Kamae I Kumagai S Meta-analysis: diagnostic accuracy of anti-cyclic citrullinated peptide antibody and rheumatoid factor for rheumatoid arthritis Ann Intern Med 2007 146 797 808 17548411 10.7326/0003-4819-146-11-200706050-00008

[B46] Janyapoon K Jivakanont P Surbrsing R Siriprapapan W Tachawuttiwat T Korbsrisate S Detection of anti-dsDNA by ELISA using different sources of antigens Pathology 2005 37 63 8 10.1080/09638280400025036 15875736

[B47] Schmidt WA Blockmans D Use of ultrasonography and positron emission tomography in the diagnosis and assessment of large-vessel vasculitis Curr Opin Rheumatol 2005 17 9 15 10.1097/01.bor.0000147282.02411.c6 15604899

[B48] Chatelain D Duhaut P Schmidt J Loire R Bosshard S Guernou M Pellet H Piette JC Sevestre H Ducroix JP Groupe de Recherche sur l'Artérite à Cellules Géantes Pathological features of temporal arteries in patients with giant cell arteritis presenting with permanent visual loss Ann Rheum Dis 2009 68 84 8 10.1136/ard.2007.084947 18252763

[B49] Schmidt WA Seifert A Gromnica-Ihle E Krause A Natusch A Ultrasound of proximal upper extremity arteries to increase the diagnostic yield in large-vessel giant cell arteritis Rheumatology (Oxford) 2008 47 96 101 10.1093/rheumatology/kem322 18077499

[B50] Edenberg J Kvambe V Sandstad E Hagen IJ Ultrasonography of temporal arteries in suspected temporal arteritis Tidsskr Nor Laegeforen 2005 125 1806 8 16012546

[B51] Schmidt WA Technology Insight: the role of color and power Doppler ultrasonography in rheumatology Nat Clin Pract Rheumatol 2007 3 35 42 quiz 59 10.1038/ncprheum0377 17203007

